# A Comparative Study of 2 Different Segmentation Methods of ADC Histogram for Differentiation Genetic Subtypes in Lower-Grade Diffuse Gliomas

**DOI:** 10.1155/2020/9549361

**Published:** 2020-09-28

**Authors:** Dan Liu, Shuai-Xiang Gao, Hong-Fan Liao, Jing-Mei Xu, Ming Wen

**Affiliations:** ^1^Department of Radiology, The Bishan Hospital of Chongqing, Bishan District, Chongqing 402760, China; ^2^Department of Hepatobiliary Surgery, The First Affiliated Hospital of Chongqing Medical University, Chongqing 400016, China; ^3^Department of Radiology, The First Affiliated Hospital of Chongqing Medical University, Chongqing 400016, China

## Abstract

**Background:**

To evaluate the diagnostic performance of apparent diffusion coefficient (ADC) histogram parameters for differentiating the genetic subtypes in lower-grade diffuse gliomas and explore which segmentation method (ROI-1, the entire tumor ROI; ROI2, the tumor ROI excluding cystic and necrotic portions) performs better.

**Materials and Methods:**

We retrospectively evaluated 56 lower-grade diffuse gliomas and divided them into three categories: IDH-wild group (IDH^wt^, 16cases); IDH mutant with the intact 1p or 19q group (IDH^mut^/1p19q^+^, 18cases); and IDH mutant with the 1p/19q codeleted group (IDH^mut^/1p19q^−^, 22cases). Histogram parameters of ADC maps calculated with the two different ROI methods: ADCmean, min, max, mode, P5, P10, P25, P75, P90, P95, kurtosis, skewness, entropy, StDev, and inhomogenity were compared between these categories using the independent *t* test or Mann–Whitney *U* test. For statistically significant results, a receiver operating characteristic (ROC) curves were constructed, and the optimal cutoff value was determined by maximizing Youden's index. Area under the curve (AUC) results were compared using the method of Delong et al.

**Results:**

The inhomogenity from the two different ROI methods for distinguishing IDH^wt^ gliomas from IDH^mut^ gliomas both showed the biggest AUC (0.788, 0.930), the optimal cutoff value was 0.229 (sensitivity, 81.3%; specificity, 75.0%) for the ROI-1 and 0.186 (sensitivity, 93.8%; specificity, 82.5%) for the ROI-2, and the AUC of the inhomogenity from the ROI-2 was significantly larger than that from another segmentation, but no significant differences were identified between the AUCs of other same parameters from the two different ROI methods. For the differentiaiton of IDH^mut^/1p19q^−^ tumors and IDH^mut^/1p19q^+^ tumors, with the ROI-1, the ADCmode showed the biggest AUC (AUC: 0.784; sensitivity, 61.1%; specificity, 90.9%), with the ROI-2, and the skewness performed best (AUC, 0.821; sensitivity, 81.8%; specificity, 77.8%), but no significant differences were identified between the AUCs of the same parameters from the two different ROI methods.

**Conclusion:**

ADC values analyzed by the histogram method could help to classify the genetic subtypes in lower-grade diffuse gliomas, no matter which ROI method was used. Extracting cystic and necrotic portions from the entire tumor lesions is preferable for evaluating the difference of the intratumoral heterogeneity and classifying IDH-wild tumors, but not significantly beneficial to predicting the 1p19q genotype in the lower-grade gliomas.

## 1. Introduction

Glioma is the most common neuroepithelial tumor in the brain which accounts for 80% of the malignant brain tumors. The severity of gliomas is further distinguished by malignant grades (I to IV) on the basis of the histopathological and clinical criteria [1]. The grade II and III gliomas are sometimes described as lower-grade gliomas, which present approximately one-third of all gliomas. Lower-grade gliomas form a biologically heterogeneous group of tumors. They are usually less aggressive tumors with a longer, indolent clinical course, but a subset of these gliomas will progress to glioblastoma (WHO grade IV gliomas) within months [2, 3]. Histology alone is often insufficient to make accurate prognostic estimates, and tumors belonging to the same WHO grade may display different malignant behavior, depending on their molecular profile. The Cancer Genome Atlas (TCGA) Analysis Working Group grouped LGGs into three robust molecular classes on the basis of mutations in isocitrate dehydrogenases 1 and 2 (IDH1 and IDH2, hereafter collectively referred to as IDH) and codeletion of chromosomes 1p and 19q. LGGs without IDH mutation are associated with the most aggressive clinical behavior and worst outcome, similar to that of glioblastomas (WHO grade IV). LGGs with IDH mutation and 1p/19q codeletion are associated with the most favorable clinical outcome and possibly improved sensitivity to procarbazine, lomustine, and vincristine chemotherapy compared with noncodeleted neoplasms. LGGs with IDH mutation and no 1p/19q codeletion are associated with an intermediate outcome, worse than those with 1p/19q codeletion, but far more favorable than IDHwt neoplasms [3]. This molecular classification has been integrated into the 2016 WHO classification of brain tumors [4].

Diffusion-weighted imaging (DWI) is a physiologic imaging modality that exploits the diffusion of water molecules to create contrast between tissues. Apparent diffusion coefficient (ADC) calculated from DWI is used as a quantitative parameter to assess the grade of restrictive diffusion and to provide information about tissue structure and cellularity [5, 6]. Previous studies have demonstrated the ability of ADC to differentiate the IDH-wild gliomas from the mutant ones, the 1p19q codeleted gliomas from those noncodeleted ones [7–9]. However, previous studies were limited to using the mean value of ADCs based on regions of interest (ROIs) from a single representative slice of a lesion or region of interest from tumor volume, which may dilute or even mask the small but important differences between different disease entities. Additionally, they may not precisely depict the tumor status due to the intrinsic heterogeneous environment of tumors. Histogram analysis of the whole lesion may offer multiple parameters containing not only the quantitative accumulated ADC parameters, such as percentiles, minimal and maximal values, and mode but also the distribution parameters, such as the kurtosis, skewness, range, StDev, inhomogenity, and entrophy, thus providing more information about the tumor heterogeneity than the mean values [10, 11]. In previous studies, two main ROI placement methods of the ADC histogram were used, including the tumor ROI excluding cystic and necrotic portions [12–14] and the entire tumor ROI containing cystic and necrotic areas [11, 15–17]. The theoretical basis of the former mentioned method is that necrotic and cystic-appearing areas may increase ADC values, which may be a confusing factor for differentiating the subtypes of gliomas based on ADC maps, but the latter method contained all compositions of the tumor, theoretically, it can better assess the heterogeneity of the tumor in its entirety. So, the purposes of this retrospective study were to evaluate whether the ADC values analyzed by the histogram method could help to classify IDH-wild tumors from IDH-mutated ones as well as IDHmut-NonCodel tumors from IDHmut-Codel ones in lower-grade diffuse gliomas and determine which segmentation method performs better.

## 2. Materials and Methods

### 2.1. Patients

We searched the electronic hospital information system and picture archiving and communication system to identify patients from January 2016 to August 2019 who met the following inclusion criteria: (1) final histopathologic results were WHO grade II–III diffuse gliomas on the basis of the WHO classification for tumors of the central nervous system; (2) diffusion-weighted MRI, T2-weighted, and postcontrast T1-weighted anatomical scan performed at initial diagnosis and prior to any surgery; (3) and known IDH1 mutation and 1p/19q codeletion status. On the other hand, patients were excluded for the poor DWI images quality, which influence the consequent image analysis. The institutional review board at the First Affiliated Hospital of Chongqing Medical University approved this study. Thus, 56 consecutive patients were included in the final study cohort. The patients were divided into the following categories: IDH wild-group (IDH^wt^), IDH mutant with the intact 1p or 19q group (IDH^mut^/1p19q^+^), and IDH mutant with the 1p/19q codeleted group (IDH^mut^/1p19q^−^).

There were 40 cases in the IDH-mutated group (18 men, 22 women; age range, 23-66 years; mean age, 41.5 ± 10.5 years; WHO grade II gliomas, *n* = 32; grade III gliomas, *n* = 8). Among the IDH-mutated group, there were 18 cases of IDH^mut^/1p19q+gliomas (8 men, 10 women; age range, 24-59 years; mean age, 40.8 ± 9.8 years; WHO grade II gliomas, *n* = 15; grade III gliomas, *n* = 3) and 22 cases of the IDH^mut^/1p19q− group (10 men, 12 women; age range, 23-66 years; mean age, 42.0 ± 11.2 years; WHO grade II glomas, *n* = 17; grade III gliomas, *n* = 5). There were 16 cases in the IDH^wt^ group (9 men, 7women; age range, 21-73 years; mean age, 51.9 ± 16.0 years; WHO grade II gliomas, *n* = 5; grade III gliomas, *n* = 11).

### 2.2. ADC Histogram Measurement

**Figure 1:**
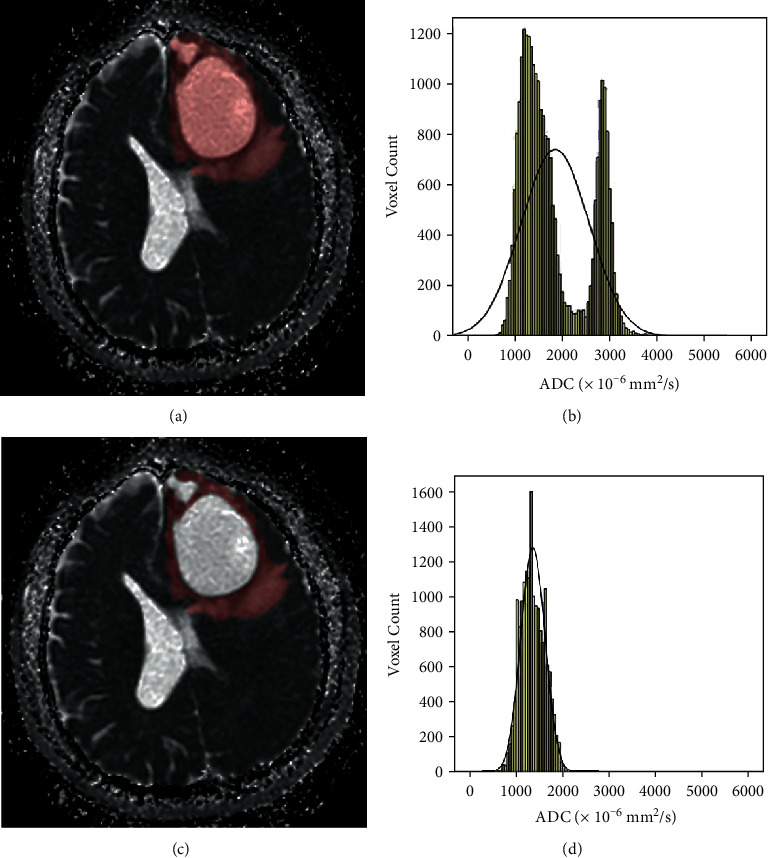
Freehand segmentation of IDH-wild LGG. (a) The ROI-1 (entire tumor ROI) delineated on an ADC map, (b) the corresponding ADC histogram of the ROI-1, (c) the ROI-2 (tumor ROI excluding cystic and necrotic portions) delineated on an ADC map, and (d) the corresponding ADC histogram of the second segmentation (color should be used for this figure).

**Figure 2:**
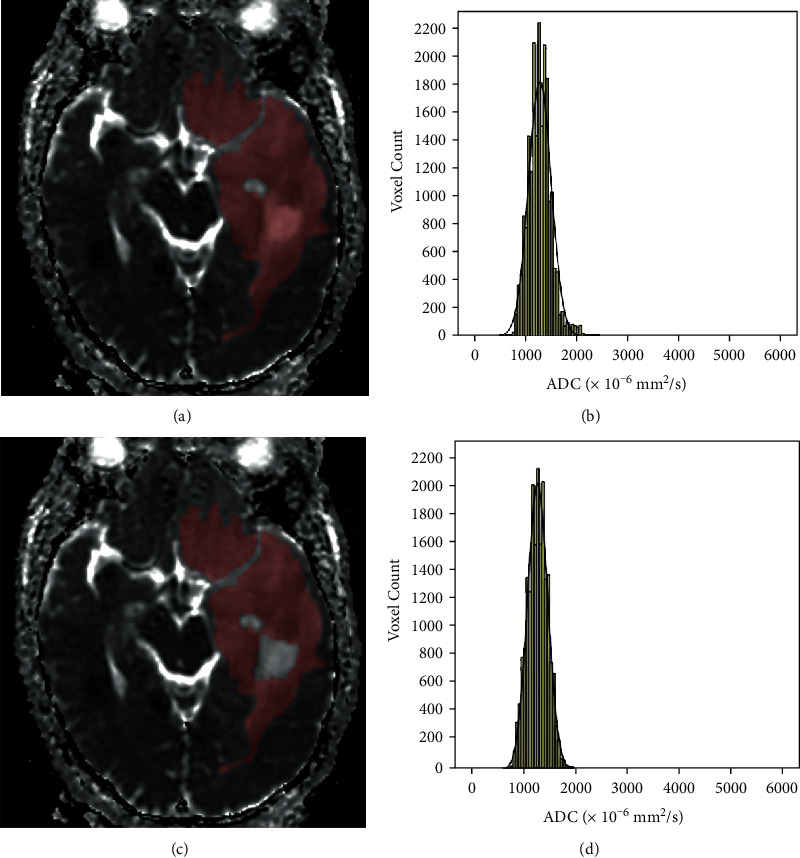
Freehand segmentation of IDH mutant with intact 1p or 19q LGG. (a) The ROI-1 delineated on an ADC map, (b) the corresponding ADC histogram of the ROI, (c) the ROI-2 delineated on an ADC map, and (d) the corresponding ADC histogram of the ROI (color should be used for this figure).

**Figure 3:**
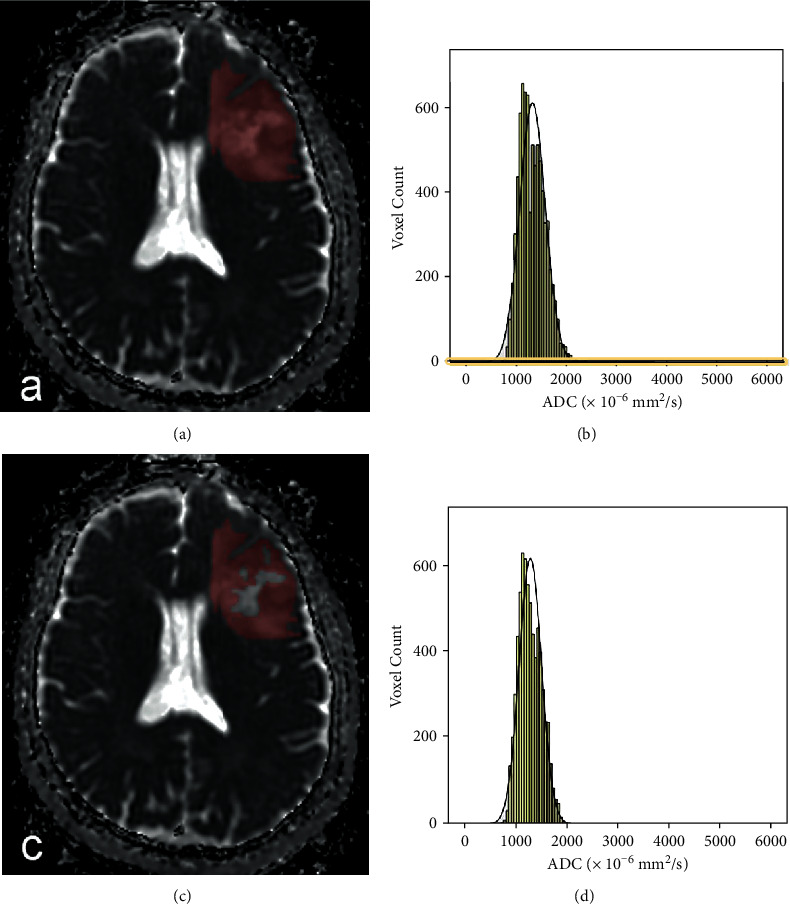
Freehand segmentation of IDH mutant with 1p/19q co- deleted LGG. (a) the ROI-1 delineated on an ADC map, (b) the corresponding ADC histogram of the ROI, (c) theROI-2 delineated on an ADC map, and (d) the corresponding ADC histogram of the ROI (color should be used for this figure).

Each case was investigated using DWI (multishot echo-planar-imaging sequence with *b* values of 0 and 1000 s/mm^2^) obtained with a 3.0 T MRI scanner (Signa HDxt, GE Medical System, WI). The ADC maps were digitally transferred from the picture archiving and communication system workstation to a personal computer and processed with an in-house software (Firevoxel, available at https://wp.nyu.edu/firevoxel/). For each case, the ROI was manually drawn by two independent radiologists with no knowledge of the final pathologic results. 2 different types of segmentation were completed: ROI-1, the entire tumor ROI containing all compositions; ROI-2, the entire tumor ROI excluding cystic and necrotic areas (cystic or necrotic portions met conditions: first, no enhancement with the contrast agent in the T1-weighted images and second, high intensity, like cerebrospinal fluid (CSF), in the T2-weighted images). According to Kang and Lue's methods, the tumor boundaries were defined with reference to the high-signal-intensity areas thought to represent the tumor tissue on the T2-weighted images [9, 11]. The ROIs were placed carefully inside the mass to avoid regions influenced by the partial volume effect, and the position of the ROIs was verified using postcontrast T1-weighted images, T2-weighted images, and T2-FLAIR imaging. Then, the following accumulated ADC parameters: mean ADC (ADCmean), maximum ADC (ADCmax), minimum ADC (ADCmin), mode ADC (ADCmode), 5th (P5 ADC), 10th (P10 ADC), 25th (P25 ADC), 75th (P75 ADC), 90th (P90 ADC), and 95th (P95 ADC) were calculated, as well as distribution parameters—the kurtosis, skewness, entropy, StDev, and inhomogenity—were also estimated. Skewness reflects the asymmetry of the distribution, being positive if more values lie to the left of the mean, and negative if the opposite. Kurtosis is a measure of the peakedness of the distribution, and a higher kurtosis indicates a sharper peak of the histogram. In case of a normal distribution, skewness equals 0 and kurtosis equals 3. Entropy, StDev, and inhomogenity represent the statistical measure of variation that can be used to characterize the image texture [13]. For further analyses of ADC histogram parameters, the results of two readers were averaged. The two different ROI segmentations and the corresponding ADC histograms are shown in Figures 1–3.

### 2.3. Histopathology and Molecular Analysis

All tissue samples underwent analysis at The First Affiliated Hospital of Chongqing Medical University's neuropathology department and center for molecular medicine testing according to the World Health Organization (WHO) 2016 guidance on immunohistochemistry testing for glioma. IDH R132H immunonegative tumors underwent multiple gene Sanger sequencing. The 1p/19q codeletion status was determined by fluorescence in situ hybridization-specific probes for the 1p36 and 19q13 loci.

### 2.4. Statistical Analysis

All statistical testing was performed with SPSS 22 (IBM) and MedClac Version15.6.1. The intraclass correlation coefficient (ICC) was calculated to evaluate interobserver agreement of all histogram parameters (*κ* = 0.00–0.20, poor correlation; *κ* = 0.21–0.40, fair correlation; *κ* = 0.41–0.60, moderate correlation; *κ* = 0.61–0.80, good correlation; *κ* = 0.81–1.00, excellent correlation) [15, 16]. The Shapiro-Wilk test was used to check whether the measurement data followed a normal distribution. Normally distributed continuous variables were compared using the independent *t* test, and nonnormally distributed continuous variables were compared using the Mann–Whitney *U* test between different molecular groups. For statistically significant results, receiver operating characteristic (ROC) curves were constructed to determine the optimal threshold for each histogram parameter to differentiate the molecular subtypes, and the optimal cutoff value was determined by maximizing Youden's index. Area under the curve (AUC) results were compared using the method of Delong et al. The results with *p* values of less than 0.05 were considered to be statistically significant.

## 3. Results

### 3.1. Comparison of Clinical Characteristics of the 56 Patients

The mean age was greater in the IDH^wt^ group than in the IDH^mut^ group (*t* = 2.422, *p* = 0.025). The proportion of grade III gliomas in the IDH^wt^ group was statistically significantly larger than in the IDH^mut^ group (Fisher's exact test *p* < 0.001). Compared with the IDH^mut^/1p19q^+^ group, neither the greater mean age or larger proportion of grade III gliomas in the IDH^mut^/1p19q^−^ group was statistically significant (*t* = −0.362, *p* = 0.716 for age, Fisher's exact test *p* = 0.709 for the proportion of grade III gliomas). The histological and molecular characteristics of the patient population are listed in Table 1.

### 3.2. Comparison of ADC Histogram Parameters for Each Method

When applying the ROI-1 segmentation, the interobserver agreements were good to excellent for all parameters in the three groups, with ICCs of 0.980-0.999, 0.868-0.999, and 0.809-0.999. With the second segmentation，the interobserver agreements were moderate to excellent for all parameters in the three groups, with ICCs of 0.880-0.987, 0.792-0.957, and 0.808-0.965.

For the IDH^wt^ tumors, the ADCmean (*p* = 0.027), P75ADC (*p* = 0.016), P90ADC (*p* = 0.009), P95ADC (*p* = 0.005), ADCmax (*p* = 0.001), StDev (*p* = 0.003), inhomogenity (*p* = 0.006), and range (*p* = 0.001) obtained from the ROI-1 were proved to be significantly greater than those from the second segmentation. Other histogram parameters did not show significant differences between the two segmentation methods (Table 2).

For the IDH^mut^/1p19q^+^ tumors, only the ADCmax (*p* = 0.044) differed significantly between the two segmentation methods, and other histogram parameters did not show significant differences between the two segmentation methods (Table 3).

For the IDH^mut^/1p19q^−^ tumors, the greater P90ADC (*p* = 0.023), P95ADC (*p* = 0.005), ADCmax (*p* = 0.004), kurtosis (*p* = 0.002), skewness (*p* = 0.001), StDev (*p* = 0.002), inhomogenity (*p* = 0.004), and range (*p* = 0.011) calculated from the ROI-1 were all statistically significant than those from the second segmentation, and other histogram parameters did not show significant differences between the two segmentation methods (Table 4).

### 3.3. Ability to Differentiate the Genetic Subtypes of Diffuse Lower-Grades Gliomas

The P75ADC (*p* = 0.017), P90ADC (*p* = 0.015), P95ADC (*p* = 0.025), ADCmax (*p* = 0.028), StDev (*p* = 0.002), inhomogenity (*p* = 0.001), and range (*p* = 0.007) from the ROI-1 were all statistically significant greater in the IDH^wt^ group than in the IDH^mut^ group, and the ADCmin (*p* = 0.006) and kurtosis (*p* = 0.007) in the IDH^wt^ group were smaller (Table 5).

The StDev (*p* < 0.001), inhomogenity (*p* < 0.001), and range (*p* = 0.004) from the ROI-2 differed significantly between IDH^wt^ gliomas and IDH^mut^ gliomas and decreased with the IDH mutation, but the ADCmin (*p* < 0.001), P5 ADC (*p* = 0.008), and kurtosis (*p* = 0.012) in the IDH^wt^ gliomas were lower than the other group (Table 5).

The ADCmean (*p* = 0.006), P5ADC (*p* = 0.008), P10ADC (*p* = 0.005), P25 ADC (*p* = 0.003), P50ADC (*p* = 0.002), P75ADC (*p* = 0.009), P90ADC (*p* = 0.048), and ADCmode (*p* = 0.002) from the ROI-1 were significantly lower in IDH^mut^/1p19q^−^gliomas, and the skewness (*p* = 0.007) was greater in IDH^mut^/1p19q^−^gliomas; the similar results were obtained from the ROI-2, but the P95 ADC (*p* = 0.019) from the ROI-2 was also significantly lower in IDH^mut^/1p19q^−^gliomas, and the kurtosis (*p* = 0.037) from the ROI-2 was lager in IDH^mut^/1p19q^−^gliomas (Table 6).

### 3.4. ROC Analysis and Comparation

ADC histogram parameters from the two different ROI methods were evaluated for their ability to discriminate the genetic subtypes using ROC analysis (Tables 7 and 8). The inhomogenity from the two different ROI methods for distinguishing IDH^wt^ gliomas from IDH^mut^ gliomas both showed the biggest AUC (0.788, 0.930), and the optimal cutoff value was 0.229 (sensitivity, 81.3%; specificity, 75.0%) for the ROI-1 and 0.186 (sensitivity, 93.8%; specificity, 82.5%) for the ROI-2, respectively. In the pairwise comparison of ROC curves with the AUC, a major finding was that the AUC of the inhomogenity from the ROI-2 was significantly larger than that from the ROI-1, but no significant differences were identified between the AUCs of other same parameters from the two different ROI methods (Table 7).

For the differentiation of IDH^mut^/1p19q^−^ tumors and IDH^mut^/1p19q^+^ tumors, the ADCmode from the ROI-1 showed the biggest AUC (AUC, 0.784; sensitivity, 61.1%; specificity, 90.9%), and the skewness from the ROI-2 showed the biggest AUC (AUC, 0.821; sensitivity, 81.8%; specificity, 77.8%), but no significant differences were identified between the AUCs of the same parameters from the two different ROI methods (Table 8).

## 4. Discussion

The results obtained from this study suggest that the ADC values analyzed by the histogram method can help to classify IDH-wild tumors from IDH-mutated tumors as well as IDHmut-Codel tumor from IDHmut-NonCodel tumors in lower-grade diffuse gliomas, no matter which ROI method is used. The StDev, inhomogenity, and range from the two different ROI methods were both larger in IDH-wild tumors compared with the IDH-mutated tumors, and the ADCmin and kurtosis in the IDH-wild group were smaller. Some different results appeared in the two different ROI methods, and one of these was that the P5ADC from the ROI-2 in the IDH-wild tumors was lower than the mutated ones, while P5ADC from ROI-1 showed no significant difference in these two types of tumors. Another difference was that the P75ADC, P90ADC, P95ADC, and ADCmax from ROI-1 were larger in IDH-wild tumors, while the same parameters from the ROI-2 showed no significant differences in the two sorts of tumor. For identifying the 1p19q-Codel ones in the IDH-mutated tumors, we found that the ADCmean, P5ADC, P10ADC, P25ADC, P50ADC, P75ADC, P90ADC, and ADCmode from the two different ROI methods were both significantly lower in the 1p19q -Codel tumors; meanwhile, the skewness was greater in this group. Compared with ROI-1, the second segmentation gave us more parameters valuable for the differentiation, such as the P95ADC and kurtosis, the P95ADC was significantly lower in the 1p19q–Codel tumors, and the kurtosis was lager in the 1p19q–Codel ones.

It is widely recognized that the pathological heterogeneity may manifest as radiologic heterogeneity on ADC maps [5], and differences in ADCs are mainly attributed to the tumor cellularity but also to the presence of necrosis or cysts [6, 11, 18]. Consequently, the ADCs within a given tumor can vary widely between different regions of that tumor [10]. The voxels with low ADC value are reportedly well correlated with highly cellular components within the tumor, which reflects tumor proliferative rate and aggressiveness [5], whereas the higher frequency of voxels with high ADC values reflect cystic, necrotic, or myxoid components. In other words, the larger intratumoral heterogeneity is the wider ADC values distribute [15]. Our results demonstrated that the IDH wild-type gliomas showed a lower ADCmin, likely representing higher cellularity and aggressiveness, consistent with some previous studies [7, 9, 19], as well as appeared larger higher end values, inhomogenity, and StDev, likely representing more common cystic and necrotic portions, which might be associated with higher grade features and larger intratumoral heterogenitiy [11, 20]; meanwhile, in the present study, we found that no matter which segmentation was used, the parameters reflecting the intratumoral heterogenitiy, such as the inhomogenity and StDev, performed better than the conventional cumulative ADC values, so it is valuable and important to evaluate the intratumoral heterogenitiy using quantitative parameters. It was interesting that the entire tumor ROI provided some additional statistically significant parameters than the other segmentation, such as the higher end ADC values. This result suggests that, to a certain degree, the necrotic and cystic components may facilitate the discrimination between IDH-mutated tumors and IDH-wild gliomas, but their performance is weaker than the inhomogenity. The inhomogenity calculated from the ROI-2 performed better than that from the ROI-1, although the diagnostic performance of the other parameters obtained from the two methods showed no statistically significant differences, which reveals that extracting cystic and necrotic portions from the entire tumor lesions is better for evaluating the difference of the intratumoral heterogeneity and more helpful to classify IDH-wild tumors in the lower-grade gliomas.

For the discrimination between the IDHmut-NonCodel tumors and the IDHmut-Codel tumors, an important finding in our study was the increase of restrictive diffusion in the IDHmut-Codel tumors. This result parallels those of previous studies reporting lower minimum ADC and a lower mean histogram ADC in tumors with 1p/19q loss compared to those without [14, 21]. There are several possible explanations for this result. One of them is the presence of calcification, which is common in IDHmut-Codel tumors [22], that may limit water content as well as hinder water movement [20], and another is that the IDHmut-Codel tumors are often highly cellular lesions with closely packed, relatively small cells in central regions and prominent secondary structure formation, which may also delay the passage of small molecules [23]. When applying the ROI-1, the ADCmode performed best at predicting the 1p19q genotype in this study, which was lower in the IDHmut-Codel tumors. ADCmode means the value that appears most frequently in a set of ADC values, and this result may also be due to the larger diffusion restriction in the IDHmut-Codel tumors. With the second segmentation, the skewness showed the biggest AUC, which was larger in IDHmut-Codel tumors. The skewness reflects the asymmetry of the ADC distribution, more pixels have lower ADC values and lie to the left of the mean of the histogram, and the skewness is more positive. Therefore, this result indicates that the IDHmut-Codel tumors contain more voxels with ADC values below the mean of the histogram, which may also be associated with the increase of restrictive diffusion, but the performance of the same parameters from the two different method showed no statistically significant differences.

As for the comparison of histogram parameters calculated from the two different segmentations, we found that almost all ADC histogram parameters calculated from the whole tumor ROIs tended to be larger than those from the other segmentation. Statistically, significant differences were found in the higher end values of cumulative ADC histograms and some histogram distribution characteristics from these two different methods in IDH-wild gliomas and IDHmut-Codel tumors, such as P75ADC, P90ADC, P95ADC, ADCmax, StDev, and inhomogenity. This result may be associated with the following reasons: the first one is that the IDH wild-type gliomas show larger intratumoral heterogeneity due to its increased cell proliferation and necrosis [7, 20], so the entire tumor ROI contained areas of necrosis and systs will lead to more higher ADC values, larger StDev, and inhomogenity. As for the codeleted tumors, the signal intensity on MR images is more heterogeneous than the noncodeleted ones [20, 24]; as a result, the StDev and inhomogenity obtained from the entire tumor ROI were lager. Conversely, the similar results of the two ROI segmentations in noncodeleted tumors can be explained by a relative homogeneity in these gliomas [20], so extracting cystic and necrotic portions from the ROIs does not cause any obvious differences.

Some limitations of this study should be considered. First, this was a retrospective study with a small study population. Therefore, the usefulness of the ADC histogram analysis should be prospectively examined in a larger and more balanced study population. Second, the ROIs were manually determined. Automatic segmentation algorithms may facilitate the procedure. Third, how well the ADC histogram analysis works in data independent from the current data was not evaluated, and our findings must be validated in further studies. Finally, it is known that the conventional ADC value derived from the monoexponential model is influenced by a combination of both diffusion and perfusion effects. Intravoxel incoherent motion (IVIM) imaging may tease out each component from the total DWI signal, so it may be more valuable to compare the ADC values derived from intravoxel incoherent motion (IVIM), but this idea needs the more widespread application of this technology.

In conclusion, our study has shown the utility of ADC histogram analysis in the characterization of the IDH-wild tumors and the IDH-mutated ones as well as the IDHmut-Codel tumors and the IDHmut-NonCodel ones in the lower-grade diffuse gliomas. Although the entire tumor ROI provided some additional statistically significant parameters than the other segmentation in the characterization of the IDH-wild tumors and the IDH-mutated ones, such as the higher end ADC values, the inhomogenity calculated from the tumor ROIs excluding cystic and necrotic portions performed best in the differentiation between these two kinds of tumors, and this suggests that extracting cystic and necrotic portions from the entire tumor lesions is needed for preferably evaluating the difference of the intratumoral heterogeneity and more helpful to classify IDH-wild tumors in the lower-grade gliomas. For predicting the 1p19q genotype in the lower-grade gliomas, when applying the entire tumor ROI, the ADCmode performed best, while applying the second method, the skewness showed the best performance, but the performance of the same parameters from the two different methods showed no statistically significant differences. Further prospective study with a more general patient population is warranted.

## Figures and Tables

**Table 1:** Comparison of clinical characteristics of the 56 patients.

	Patient group			
	IDH^wt^	IDH^mut^		
		Total	IDH^mut^/1p19q^+^	IDH^mut^/1p19q^−^
WHO grade				
II	5	32	15	17
III	11	8	3	5
Total	16^#^	40^#^	18^##^	22^##^
Age (years, *X* ± *SD*)	51.9 ± 16.0^∗^	41.5 ± 10.5^∗^	40.8 ± 9.8^∗∗^	42.0 ± 11.2^∗∗^

^#^ *Pearson*'s chi-square test *p* < 0.001, ^##^*Fisher*'s exact test *p* = 0.709; ^∗^*t* = 2.422, *p* = 0.025; ^∗∗^*t* = −0.362, *p* = 0.716.

**Table 2:** ADC histogram parameters of the IDH^wt^ tumors using different ROI methods.

Values (×10^−6^ mm^2^/s)	The tumor ROI	The tumor ROI excluding cystic and necrotic portions	Test values	*p* values
ADCmean	1646.10 ± 402.18	1368.54 ± 254.39	-2.333^a^	0.027
ADCmin	518.50 ± 226.12	485.38 ± 195.34	-0.443^a^	0.661
P5 ADC	972.53 ± 207.95	880.19 ± 168.79	-1.379^a^	0.178
P10 ADC	1066.38 ± 239.79	966.75 ± 184.50	-1.317^a^	0.198
P25 ADC	1281.41 ± 306.36	1142.50 ± 220.39	-1.472^a^	0.151
P50 ADC	1606.94 ± 442.95	1368.63 ± 267.23	-1.843^a^	0.075
P75 ADC	2022.41 ± 580.95	1593.25 ± 304.42	-2.617^a^	0.016
P90 ADC	2298.56 ± 620.70	1767.56 ± 331.19	-2.848^a^	0.009
P95 ADC	2403.84 ± 609.61	1864.00 ± 342.97	-3.085^a^	0.005
ADCmax	3606.75 ± 1159.31	2450.25 ± 417.17	-3.755^a^	0.001
ADCmode	1616.19 ± 583.11	1517.06 ± 533.69	-0.502^a^	0.620
Kurtosis	-0.527 (-1.027, 0.480)	−0.401 ± 0.429	-0.075^b^	0.956
Skewness	0.434 ± 0.689	0.065 ± 0.310	-1.957^a^	0.060
StDev	388.630 (343.670, 689.601)	303.055 ± 72.511	-2.902^b^	0.003
Inhomogenity	0.278 ± 0.069	0.220 ± 0.033	-3.047^a^	0.006
Range	3087.31 ± 1130.76	1966.56 ± 389.39	-3.799^a^	0.001
Entropy	3.968 ± 0.224	4.082 ± 0.199	-1.527^a^	0.137

^a^ *t* value; ^b^*Z* value.

**Table 3:** ADC histogram parameters for IDH^mut^/1p19q^+^ tumors using different ROI methods.

Values (×10^−6^ mm^2^/s)	The tumor ROI	The tumor ROI excluding cystic or necrotic portions	Test values	*p* values
ADCmean	1575.71 ± 286.14	1511.41 ± 253.75	0.713^a^	0.481
ADCmin	740.69 ± 123.11	751.83 ± 131.62	-0.26^a^	0.795
P5 ADC	1085.02 ± 149.95	1077.67 ± 151.22	0.147^a^	0.884
P10 ADC	1176.92 ± 179.65	1163.22 ± 177.72	0.230^a^	0.820
P25 ADC	1355.22 ± 235.42	1332.89 ± 232.28	0.287^a^	0.776
P50 ADC	1560.53 ± 286.86	1526.11 ± 280.79	0.364^a^	0.718
P75 ADC	1805.81 ± 430.58	1687.00 ± 301.50	0.959^a^	0.344
P90 ADC	1981.53 ± 471.58	1811.67 ± 307.63	1.280^a^	0.209
P95 ADC	2096.78 ± 485.56	1879.68 ± 302.98	1.609^a^	0.117
ADCmax	2957.83 ± 1009.01	2415.72 ± 362.81	2.145^a^	0.044
ADCmode	1571.61 ± 349.80	1575.94 ± 355.41	-0.037^a^	0.971
Kurtosis	-0.342 (-0.518, 1.988)	-0.354 (-0.482, 0.080)	-0.253^b^	0.800
Skewness	-0.200 (-0.446, 0.912)	-0.227 (-0.526, 0.183)	-0.886^b^	0.376
StDev	288.203 (215.733, 366.08)	248.611 ± 58.938	-1.487^b^	0.137
Inhomogenity	0.172 (0.155, 0.244)	0.163 ± 0.025	-1.361^b^	0.173
Range	2232.56 ± 1076.62	1688.11 ± 406.17	2.007^a^	0.057
Entropy	3.970 ± 0.281	4.054 ± 0.246	-0.944^a^	0.352

^a^, *t* value; ^b^, Z value.

**Table 4:** ADC histogram parameters for IDH^mut^/1p19q^−^ tumors using different ROI methods.

Values(×10^−6^ mm^2^/s)	The tumor ROI	The tumor ROI excluding cystic or necrotic portions	Test values	*p* values
ADCmean	1353.09 ± 131.59	1296.48 ± 128.26	-1.445^a^	0.156
ADCmin	670.09 ± 116.37	672.82 ± 125.13	-0.075^a^	0.941
P5 ADC	974.11 ± 70.45	966.09 ± 69.62	-0.380^a^	0.706
P10 ADC	1033.61 ± 77.99	1024.23 ± 81.31	-0.391^a^	0.698
P25 ADC	1152.87 ± 101.56	1137.09 ± 100.86	-0.518^a^	0.607
P50 ADC	1306.20 ± 131.69	1276.00 ± 129.73	-0.766^a^	0.448
P75 ADC	1499.43 ± 159.83	1432.64 ± 153.36	-1.414^a^	0.165
P90 ADC	1728.45 ± 226.92	1585.68 ± 170.46	-2.360^a^	0.023
P95 ADC	1925.86 ± 344.50	1677.45 ± 180.02	-2.998^a^	0.005
ADCmax	2776.43 ± 743.58	2220.59 ± 367.02	-3.144^a^	0.004
ADCmode	1158.50 (1093.50, 1329.88)	1170.50 (1093.50, 1328.25)	-0.094^b^	0.925
Kurtosis	1.191 (0.242, 2.290)	0.102(-0.309, 0.328)	-3.157^b^	0.002
Skewness	0.984 ± 0.646	0.378 (0.231, 0.642)	-3.898^b^	0.001
StDev	293.603 ± 97.224	217.231 ± 43.743	-3.360^a^	0.002
Inhomogenity	0.197 (0.165,0.251)	0.166 ± 0.025	-2.900^b^	0.004
Range	1939.25 (1391.00, 2733.13)	1547.77 ± 405.23	-2.547^b^	0.011
Entropy	3.933 ± 0.225	3.950 ± 0.249	1.450^a^	0.155

^a^ *t* value; ^b^*Z* value.

**Table 5:** Comparison of ADC histogram parameters of the two different ROIs between IDH^wt^ gliomas and IDH^mut^ gliomas.

Values (×10^−6^ mm^2^/s)	IDH^wt^ group, *n* = 16	IDH^mut^ group, *n* = 40	Test values	*p* values
ADCmean^1^	1646.10 ± 402.18	1419.95 (1279.30, 1565.89)	-1.796^b^	0.073
ADCmean^2^	1368.54 ± 254.39	1341.13 (1242.53, 1547.90)	-0.109^b^	0.913
ADCmin^1^	518.50 ± 226.12	701.86 ± 123.14	-3.067^a^	0.006
ADCmin^2^	485.38 ± 195.34	708.38 ± 132.54	-4196^a^	<0.001
P5 ADC^1^	972.53 ± 207.95	1010.00 (929.86, 1094.88)	-0.852^b^	0.394
P5 ADC^2^	880.19 ± 168.79	1009.00 (913.75, 1076.75)	-2.648^b^	0.008
P10 ADC^1^	1066.38 ± 239.79	1073.25 (987.88, 1161.63)	-0.381^b^	0.703
P10 ADC^2^	966.75 ± 184.50	1069.50 (970.75, 1173.75)	-1.904^b^	0.057
P25 ADC^1^	1281.41 ± 306.36	1205.75 (1104.88, 1336.38)	-0.689^b^	0.491
P25 ADC^2^	1142.50 ± 220.39	1178.50 (1081.50, 1331.75)	-1.904^b^	0.301
P50 ADC^1^	1606.94 ± 442.95	1352.75 (1255.63, 1586.38)	-1.560^b^	0.119
P50 ADC^2^	1368.63 ± 267.23	1320.50 (1225.00, 1547.25)	-01.109^b^	0.913
P75 ADC^1^	2022.41 ± 580.95	1586.50 (1411.63, 1764.75)	-2.385^b^	0.017
P75 ADC^2^	1593.25 ± 304.42	1481.50 (1356.50, 1749.00)	-0.580^b^	0.562
P90 ADC^1^	2298.56 ± 620.70	1815.25 (1615.50, 2003.50)	-2.430^b^	0.015
P90 ADC^2^	1767.56 ± 331.19	1687.38 ± 264.32	0.953^a^	0.345
P95 ADC^1^	2403.84 ± 609.61	2002.78 ± 417.24	2.415^a^	0.025
P95 ADC^2^	1864.00 ± 342.97	1768.46 ± 260.47	1.135^a^	0.261
ADCmax^1^	3606.75 ± 1159.31	2716.50 (2161.63, 3409.75)	-2.195^b^	0.028
ADCmax^2^	2450.25 ± 417.17	2308.40 ± 373.60	1.242^a^	0.220
ADCmode^1^	1616.19 ± 583.11	1285.00 (1139.50, 1619.25)	-1.324^b^	0.185
ADCmode^2^	1517.06 ± 533.69	1273.50 (1142.25, 1610.25)	-0.517^b^	0.605
Kurtosis^1^	-0.527 (-1.027, 0.480)	0.661 (-0.397, 2.273)	-2.721^b^	0.007
Kurtosis^2^	−0.401 ± 0.429	-0.126 (-0.374, 0.304)	-2.512^b^	0.012
Skewness^1^	0.434 ± 0.689	0.664 ± 0.831	-0.979^a^	0.332
Skewness^2^	0.065 ± 0.310	0.198 ± 0.522	-0.955^a^	0.344
StDev^1^	388.630 (343.670, 689.60)	278.864 (217.080, 357.755)	-3.156^b^	0.002
StDev^2^	303.055 ± 72.511	231.352 ± 51.862	4.110^a^	<0.001
Inhomogenity^1^	0.278 ± 0.069	0.190 (0.160, 0.231)	-3.347^b^	0.001
Inhomogenity^2^	0.220 ± 0.033	0.165 ± 0.025	6.048^a^	<0.001
Range^1^	3087.31 ± 1130.76	1941.25 (1406.00, 2788.38)	-2.721^b^	0.007
Range^2^	1966.56 ± 389.39	1610.93 ± 406.61	2.991^a^	0.004
Entropy^1^	3.968 ± 0.224	3.950 ± 0.249	0.218^a^	0.825
Entropy^2^	4.082 ± 0.199	4.034 ± 0.203	0.798^a^	0.428

^1^ROI-1; ^2^ROI-2; ^a^*t* value; ^b^*Z* value.

**Table 6:** Comparison of ADC histogram parameters of the two different ROIs between the IDH^mut^/1p19q^−^ group and IDH^mut^/1p19q^+^ group.

Values (×10^−6^ mm^2^/s)	IDH^mut^/1p19q^+^, *n* = 18	IDH^mut^/1p19q^−^, *n* = 22	Test values	*p* value
ADCmean^1^	1575.71 ± 286.14	1353.09 ± 131.59	3.048^a^	0.006
ADCmean^2^	1511.411 ± 253.75	1296.48 ± 128.26	3.268^a^	0.003
ADCmin^1^	740.69 ± 123.11	670.09 ± 116.37	1.860^a^	0.071
ADCmin^2^	751.83 ± 131.62	672.82 ± 125.13	1.941^a^	0.060
P5 ADC^1^	1085.02 ± 149.95	974.11 ± 70.45	2.888^a^	0.008
P5 ADC^2^	1077.67 ± 151.22	966.09 ± 69.62	2.890^a^	0.008
P10 ADC^1^	1176.92 ± 179.65	1033.61 ± 77.99	3.150^a^	0.005
P10 ADC^2^	1163.22 ± 177.72	1024.23 ± 81.31	3.066^a^	0.006
P25 ADC^1^	1355.22 ± 235.42	1152.87 ± 101.56	3.397^a^	0.003
P25 ADC^2^	1332.89 ± 232.28	1137.09 ± 100.86	3.329^a^	0.003
P50 ADC^1^	1560.53 ± 286.86	1306.20 ± 131.69	3.474^a^	0.002
P50 ADC^2^	1526.11 ± 280.79	1276.00 ± 129.73	3.487^a^	0.002
P75 ADC^1^	1805.81 ± 430.58	1499.43 ± 159.83	2.862^a^	0.009
P75 ADC^2^	1687.00 ± 301.50	1432.64 ± 153.36	3.252^a^	0.003
P90 ADC^1^	1981.53 ± 471.58	1728.45 ± 226.92	2.088^a^	0.048
P90 ADC^2^	1811.67 ± 307.63	1585.68 ± 170.46	2.786^a^	0.010
P95 ADC^1^	2096.78 ± 485.56	1925.86 ± 344.50	1.300^a^	0.201
P95 ADC^2^	1879.68 ± 302.98	1677.45 ± 180.02	2.494^a^	0.019
ADCmax^1^	2957.83 ± 1009.01	2776.43 ± 743.58	0.654^a^	0.517
ADCmax^2^	2415.72 ± 362.81	2220.59 ± 367.02	1.681^a^	0.101
ADCmode^1^	1571.61 ± 349.80	1158.50 (1093.50, 1329.88)	-3.059^b^	0.002
ADCmode^2^	1575.94 ± 355.41	1170.50 (1093.50, 1328.25)	-3.086^b^	0.002
Skewness^1^	-0.200 (-0.446, 0.912)	0.984 ± 0.646	-2.644^b^	0.007
Skewness^2^	-0.227 (-0.526, 0.183)	0.378 (0.231, 0.642)	-2.093^b^	<0.001
Kurtosis^1^	-0.342 (-0.518, 1.988)	1.191 (0.242, 2.290)	-1.930^b^	0.054
Kurtosis^2^	-0.354 (-0.482, 0.080)	0.102 (-0.309, 0.328)	-3.453^b^	0.037
StDev^1^	288.203 (215.736, 366.088)	293.603 ± 97.224	-0.214^b^	0.828
StDev^2^	248.611 ± 58.938	217.231 ± 43.743	1.932^a^	0.061
Inhomogenity^1^	0.172 (0.155, 0.244)	0.197 (0.165, 0.251)	-1.142^b^	0.253
Inhomogenity^2^	0.163 ± 0.025	0.166 ± 0.025	-0.346^a^	0.731
Range^1^	2232.56 ± 1076.52	1939.25 (1391.00, 2733.13)	-0.381^b^	0.703
Range^2^	1688.11 ± 406.17	1547.77 ± 405.23	1.089^a^	0.283
Entropy^1^	3.970 ± 0.281	3.933 ± 0.225	0.467^a^	0.643
Entropy^2^	4.054 ± 0.246	3.950 ± 0.249	0.527^a^	0.601

^1^ROI-1; ^2^ROI-2; ^a^*t* value; ^b^*Z* value.

**Table 7:** ROC Curve analysis of different parameters for distinguishing IDH^wt^ gliomas from IDH^mut^ gliomas.

Parameter	ROI	AUC	Specificity (%)	Sensitivity (%)	Cutoff value	*p* value
ADCmin	ROI-1	0.749	87.5	62.5	560.00	0.180
	ROI-2	0.831	90.0	62.5	543.00	

P5 ADC	ROI-1	—	—	—	—	—
	ROI-2	0.728	57.5	81.2	980.00	

P75 ADC	ROI-1	0.705	68.8	70.0	1688.25	—
	ROI-2	—	—	—	—	

P90 ADC	ROI-1	0.709	68.8	77.5	1962.00	—
	ROI-2	—	—	—	—	

P95 ADC	ROI-1	0.709	68.8	72.5	2164.75	—
	ROI-2	—	—	—	—	

ADCmax	ROI-1	0.689	81.3	57.5	2849.00	—
	ROI-2	—	—	—	—	

Kurtosis	ROI-1	0.734	87.5	56.2	-0.487	0.826
	ROI-2	0.716	47.5	87.5	-0.042	

StDev	ROI-1	0.772	87.5	65.0	321.702	0.646
	ROI-2	0.803	68.8	80.0	272.351	

Inhomogenity	ROI-1	0.788	81.3	75.0	0.229	0.013
	ROI-2	0.930	93.8	82.5	0.186	

Range	ROI-1	0.734	81.3	60.0	2144.5	0.838
	ROI-2	0.747	68.8	82.5	1977.5	

**Table 8:** ROC curve analysis of different parameters for distinguishing IDH^mut^/1p19q^−^ tumors and IDH^mut^/1p19q^+^ tumors.

Parameter	ROI	AUC	Specificity (%)	Sensitivity (%)	Cutoff value	*p* value
ADCmean	ROI-1	0.715	55.6	95.5	1546.32	0.151
	ROI-2	0.758	72.2	81.8	1387.97	

P5 ADC	ROI-1	0.735	50.0	100.0	1108.00	1.00
	ROI-2	0.735	44.4	100.0	1110.00	

P10 ADC	ROI-1	0.749	50.0	100.0	1177.50	1.00
	ROI-2	0.749	50.0	100.0	1191.00	

P25ADC	ROI-1	0.766	66.7	86.4	1246.75	1.00
	ROI-2	0.766	50.0	100.0	1345.00	

P50 ADC	ROI-1	0.763	55.6	100.0	1581.25	1.00
	ROI-2	0.763	67.7	86.4	1384.00	

P75 ADC	ROI-1	0.710	50.0	100.0	1788.50	1.00
	ROI-2	0.710	66.7	81.8	1540.00	

P90 ADC	ROI-1	0.669	44.4	90.9	1984.00	1.00
	ROI-2	0.669	55.6	90.9	1812.50	

P95ADC	ROI-1	—	—	—	—	—
	ROI-2	0.689	61.1	86.4	1979.00	

ADCmode	ROI-1	0.784	61.1	90.9	1448.75	0.729
	ROI-2	0.787	61.1	90.9	1450.00	

Skewness	ROI-1	0.747	100.0	44.4	-0.164	0.178
	ROI-2	0.821	81.8	77.8	0.186	

Kurtosis	ROI-1	—	—	—	—	—
	ROI-2	0.694	86.4	55.6	-0.349	

## Data Availability

The clinical data used to support the findings of this study were provided by Department of Radiology, the First Affiliated Hospital of Chongqing Medical University, and cannot be made freely available. Access to these data will be considered by the author upon request, with permission from the Director of the Department of Radiology of this hospital.

## Additional Points

*Highlights*. (i) Lower-grade gliomas form a biologically heterogeneous group of tumors, whose clinical behavior, outcome, and therapeutic sensitivity are, to some extent, depending on their molecular profile. (ii) ADC values analyzed by the histogram method could help to classify IDH-wild tumors from IDH-mutated tumors as well as IDHmut-Codel tumors from IDHmut-NonCodel tumors in lower-grade diffuse gliomas. (iii) Extracting cystic and necrotic portions from the entire tumor lesions can preferably evaluate the difference of the intratumoral heterogeneity and classifying IDH-wild ones in the lower-grade gliomas.
